# Supplementary material for: The causal relationship between the human gut microbiota and pyogenic arthritis: a Mendelian randomization study
Source: Front Cell Infect Microbiol. 2024 Nov 26;14:1452480. doi: 10.3389/fcimb.2024.1452480 (PMC11629706; doi:10.3389/fcimb.2024.1452480)
Supplement: Supplementary file 1 [file DataSheet1.zip › mendelian test/ebi-a-GCST90027487.csv_scatter.pdf]

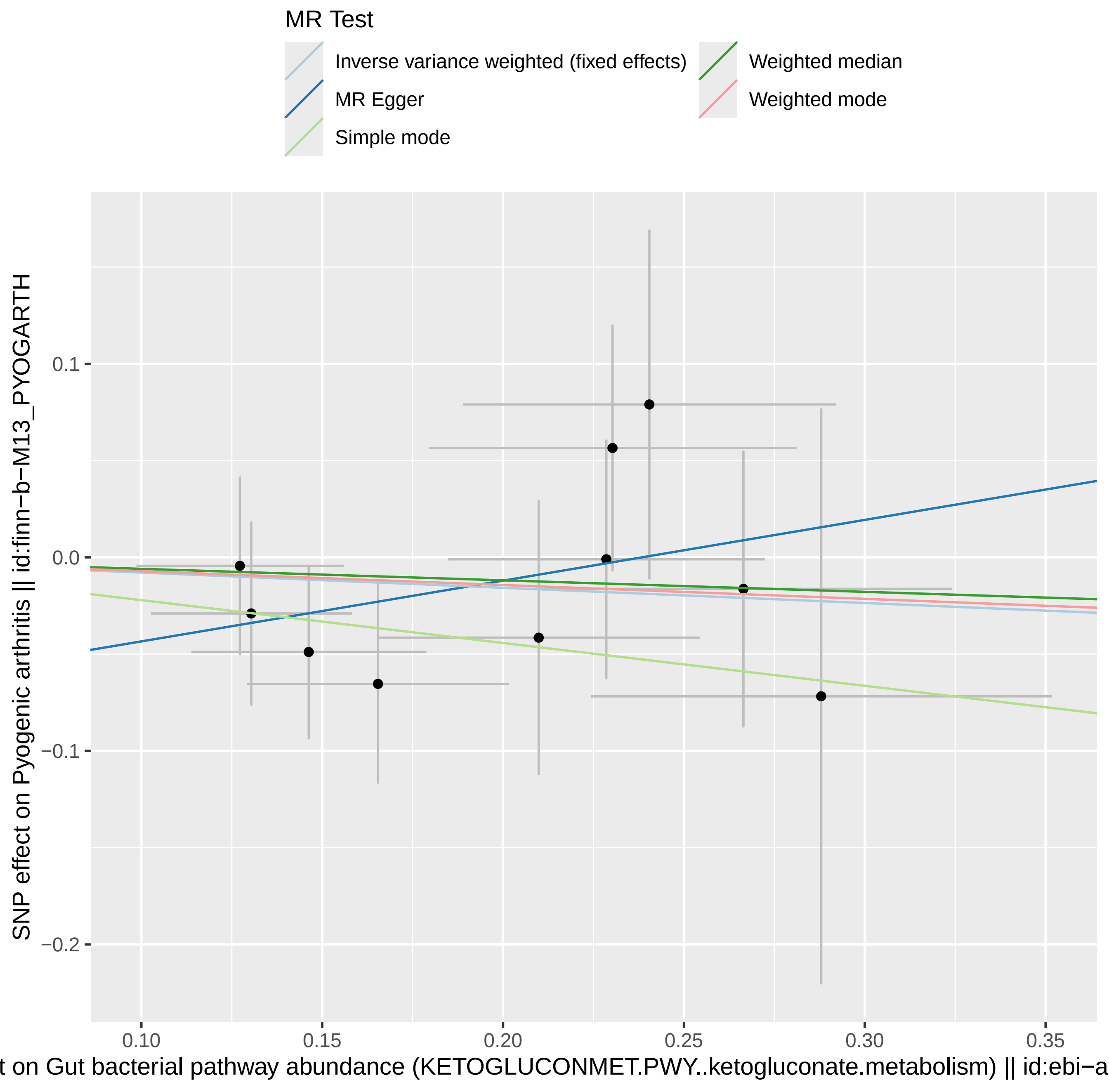

MR Test
Inverse variance weighted (fixed effects)
MR Egger
Simple mode
Weighted median
Weighted mode
0.1
0.0
−0.1
−0.2
0.10
0.15
0.20
0.25
0.30
0.35
SNP effect on Pyogenic arthritis || id:finn−b−M13_PYOGARTH
t on Gut bacterial pathway abundance (KETOGLUCONMET.PWY..ketogluconate.metabolism) || id:ebi−a
